# Inoculation With *Piriformospora indica* Is More Efficient in Wild-Type Rice Than in Transgenic Rice Over-Expressing the Vacuolar H^+^-PPase

**DOI:** 10.3389/fmicb.2019.01087

**Published:** 2019-05-15

**Authors:** Amanda Azevedo Bertolazi, Sávio Bastos de Souza, Katherine Fraga Ruas, Eliemar Campostrini, Carlos Eduardo de Rezende, Cristina Cruz, Juliana Melo, Carlos Moacir Colodete, Ajit Varma, Alessandro Coutinho Ramos

**Affiliations:** ^1^Laboratory of Environmental Microbiology and Biotechnology, Universidade Vila Velha (UVV), Vila Velha, Brazil; ^2^Laboratory of Plant Physiology, CCTA, Universidade Estadual do Norte Fluminense (UENF), Campos dos Goytacazes, Brazil; ^3^Laboratory of Environmental Sciences, CBB, Universidade Estadual do Norte Fluminense (UENF), Campos dos Goytacazes, Brazil; ^4^Centre for Ecology, Evolution and Environmental Changes (Ce3C), Faculty of Sciences, Universidade de Lisboa, Campo Grande, Portugal; ^5^Amity Institute of Microbial Technology, Amity University, Noida, India

**Keywords:** endophytic fungus, *Oryza sativa*, plant growth, photosynthesis, H^+^ pumps, H^+^-PPase

## Abstract

Achieving food security in a context of environmental sustainability is one of the main challenges of the XXI century. Two competing strategies to achieve this goal are the use of genetically modified plants and the use of plant growth promoting microorganisms (PGPMs). However, few studies assess the response of genetically modified plants to PGPMs. The aim of this study was to compare the response of over-expressing the vacuolar H^+^-PPase (AVP) and wild-type rice types to the endophytic fungus; *Piriformospora indica*. *Oryza sativa* plants (WT and AVP) were inoculated with *P. indica* and 30 days later, morphological, ecophysiological and bioenergetic parameters, and nutrient content were assessed. AVP and WT plant heights were strongly influenced by inoculation with *P. indica*, which also promoted increases in fresh and dry matter of shoot in both genotypes. This may be related with the stimulatory effect of *P. indica* on ecophysiological parameters, especially photosynthetic rate, stomatal conductance, intrinsic water use efficiency and carboxylation efficiency. However, there were differences between the genotypes concerning the physiological mechanisms leading to biomass increment. In WT plants, inoculation with *P. indica* stimulated all H^+^ pumps. However, in inoculated AVP plants, H^+^-PPase was stimulated, but P- and V-ATPases were inhibited. Fungal inoculation enhanced nutrient uptake in both shoots and roots of WT and AVP plants, compared to uninoculated plants; but among inoculated genotypes, the nutrient uptake was lower in AVP than in WT plants. These results clearly demonstrate that the symbiosis between *P. indica* and AVP plants did not benefit those plants, which may be related to the inefficient colonization of this fungus on the transgenic plants, demonstrating an incompatibility of this symbiosis, which needs to be further studied.

## Introduction

Recent decades have been marked by important changes in the agricultural production, due to the larger use of fertilizers (Clay, 2013). Total consumption of N, P, and K fertilizers increased from 30 million tons per year (Mt/year) in 1960 to 193 Mt/year in 2014, a 500% increase (FAO, 2017), which has been contributing to food security while creating complex environmental issues (Thilagar et al., 2016). In this context, the concept of sustainable agriculture has become of utmost relevance (Wezel et al., 2014), and new technologies that optimize the balance between crop yield and environment impact are being developed (Long et al., 2015).

Genetic engineering has improved several crop characteristics such as tolerance to drought (Todaka et al., 2015) and salt (Cabello et al., 2014; Roy et al., 2014), nutrient use efficiency (Han et al., 2015), insect resistance (Birkett and Pickett, 2014), and pathogen resistance (Boyd et al., 2013).

The membrane-bound proton-pumping pyrophosphatase (V-PPase), together with the V-type H^+^-ATPase, generate the proton motive force that drives vacuolar membrane solute transport (Graus et al., 2018). Transgenic plants expressing the vacuolar proton-pumping pyrophosphatase (H^+^-PPase) gene (AVP1) have enhanced shoot and root biomass production under various abiotic stress conditions, including low nutrient availability, drought and salinity (Schilling et al., 2017). Several mechanisms have been proposed to explain the greater size of plants over-expressing AVP1, including vacuolar acidification (Gaxiola et al., 2001) and regulation of cytoplasmic inorganic pyrophosphate (PPi) concentrations (Ferjani et al., 2011). Vacuolar acidification occurs due increased proton transport by both H^+^-PPase and the vacuolar H^+^-ATPase (V-H^+^-ATPase) (Kriegel et al., 2015). Although usually viewed as a tonoplast resident proton pump responsible for acidifying the vacuole, AVP1 also contributes to transport, probably by mediating the trafficking of plasma membrane (PM) H^+^-ATPase and associated proteins, including PIN1. Changes in intra-cellular auxin levels are also known to alter the expression of P-H^+^-ATPase genes, establishing a feedback loop whereby AVP1 activity can regulate both targeting and level of the PM proton pump. AVP1 has a recognized role in facilitating auxin transport and regulating auxin-related developmental processes (Li et al., 2005).

In addition to genetically modified plants, plant growth promoting microorganisms (PGPM) offer promising and environmentally friendly strategies for conventional and organic agriculture worldwide (Berg, 2009). Among PGPM, the root endophytic fungus *Piriformospora indica*, a basidiomycete of the order Sebacinales (Verma et al., 1998, Verma et al., 1999) is one of the most promising. This fungus has growth-promoting effects on a broad range of plants, including important agricultural cultures such as barley, rice and wheat, medicinal plants such as *Aloe vera* and *Artemisia annua*, and model plants such as *Arabidopsis thaliana* and tobacco (Gill et al., 2016).

The ubiquity and easy culture of *P. indica* in axenic conditions, along with its ability to improve plant growth and protect it against biotic and abiotic stress, make this fungus an efficient microsymbiont to improve crop production and yield in sustainable agriculture, floriculture and agroforestry systems. However, the success of any microbial inoculum under field conditions should be tested for each case, since the effectiveness of symbiosis depends on complex interactions between plants, microsymbionts, and the environment (Franken, 2012; Bashan et al., 2014). The molecular and physiological bases of the mechanisms underlying the association between crop plants and *P. indica* are being intensively studied. In fact, Lee et al. (2011) correlated plant growth promotion of *P. indica* and alterations in root morphology of Chinese cabbage with the up-regulation of genes encoding a vacuolar-type H^+^-ATPase, a pyrophosphate-energized vacuolar membrane proton pump and the auxin influx carrier AUX1.

In light of the literature, it is possible to observe an analogy between the benefits provided by PGPM and transgenic plants with overexpression of the type I H^+^-PPase AVP1, since both stimulate plant growth and biomass production through similar molecular mechanisms (Gaxiola et al., 2011; Varma et al., 2012). In this work we investigated the effect of *P. indica* on transgenic rice plants overexpressing vacuolar H^+^-PPases through a multidimensional analysis, considering morphological, ecophysiological, biochemical and nutritional characteristics.

## Materials and Methods

### Culture of the Endophytic Fungus

The endophytic fungus *Piriformospora indica* was obtained from the culture collection of the Centre for Ecology, Evolution and Environmental Changes, at the Science Faculty, University of Lisbon, Portugal.

The stock cultures were propagated as described by Varma et al. (1999). In order to propagate the fungus in liquid medium, 10 fungal plugs (1 cm diameter) were removed from de edges of active colonies and inoculated in Erlenmeyer flasks (250 mL), which contained 50 mL of modified Kafer medium (Hill and Kaefer, 2001), composed of: peptone 2 g⋅L^-1^, casein 1 g⋅L^-1^, yeast extract 1 g⋅L^-1^, glucose 10 g⋅L^-1^, 50 mL⋅L^-1^ of macronutrients solution (NaNO_3_ 12 g⋅L^-1^, KCl 10.4 g⋅L^-1^, MgSO_4_⋅7H_2_O 10.4 g⋅L^-1^, and KH_2_PO_4_ 30.4 g⋅L^-1^), 10 mL⋅L^-1^ of micronutrients solution [H_3_BO_3_ 13.3 μM, MnCl_2_⋅4H_2_O 7 μM, ZnSO_4_⋅7H_2_O 2 μM, CuSO_4_⋅5 H_2_O 0,5 μM, (NH_4_)_6_Mo_7_O_24_⋅4H_2_O] 0.086 μM, Fe-EDTA 1 mL⋅L^-1^, 1 mL⋅L^-1^ of vitamin solution, agar 20 g⋅L^-1^. Medium pH was adjusted to 6.5 before sterilization at 121°C for 40 min. The fungus was incubated in shakers at 28 ± 2°C and 125 rpm for 10 days, under continuous illumination (80 μmol⋅m^-2^⋅s^-1^) (Das et al., 2012).

### Plant Materials and Experimental Design

WT and AVP1OX rice (*Oryza sativa*) seeds were obtained from Arizona University, United States. Seeds were surface-sterilized in 70% ethanol for 3 min, in 2% sodium hypochlorite solution for 5 min, rinsed several times in sterile water, and then planted into trays (5 L) filled with sterile sand. Autoclaving was carried out twice, with a 24 h interval in between, at 121°C for 60 min. Plants were grown in a greenhouse [30% interception of photosynthetic active radiation, maximum photosynthetic photon flux density (PPFD) ≅ 1400 μmol⋅m^-2^⋅s^-1^] (Campos dos Goytacazes, Rio de Janeiro, Brazil, Latitude: 21° 45^′^ 15^′′^ S, Longitude: 41° 19^′^ 28^′′^ W), for 30 days. The seedlings (WT and AVP1OX) were then transplanted into plastic pots (5 L, 10 seedlings per pot, three biological replicates to each treatment) filled with a 2:1 sterile mixture of sand: dystrophic red-yellow latosol.

For inoculation, the mycelium was filtered through cheesecloth to remove the excess medium and washed three times with distilled water. After each washing step, the mycelium was collected by centrifugation at 4,000 *g* for 7 min. The pellet was suspended in distilled water and the inoculum (1% w/v) of crushed mycelium of *P. indica* was irrigated around the seedlings’ roots. The mock-treated seedlings were only irrigated with sterile distilled water. Uninoculated and inoculated WT and AVP1OX plants were grown in a greenhouse (30% interception of photosynthetic active radiation), for another 30 days. Plants were watered, three times a week, with modified Clark solution (½ strength), pH 5.5–5.6 (Clark, 1975). All analyses were performed 30 days after inoculation (in 60 day-old plants).

### Growth Parameters and Root Colonization

Thirty days after inoculation plants were harvested, and shoot and root fresh and dry weight, shoot height, shoot water content and fungal root colonization were determined. Plant material dried at 60°C until constant weight was used to obtain plant biomass. Shoot water content was determined by using the following formula: Shoot water content = [(shoot fresh matter-shoot dry matter)/shoot dry matter × 100]. Fungal colonization was determined according to Phillips and Hayman (1970) with modifications. Root samples were cleaned by heating in 10% KOH solution for 60 min, then rinsed in water and stained by simmering in 0.02% trypan blue for 20 min. After, the excess stain was removed in 50% lactophenol for 1–2 h prior to observation. Twenty root segments of 1 cm length were randomly chosen from each set and examined under a light microscope. The distribution of chlamydospores within the root cortex was taken as an index of colonization. The percentage colonization was determined using the following formula: root colonization (%) = [(number of colonized segments/total number of segments examined) × 100]. Colonization effectiveness was calculated by the formula: colonization effectiveness (%) = [(Total shoot dry weight of inoculated plants) - (Total shoot dry weight of uninoculated plants)/(Total shoot dry weight of uninoculated plants)] × 100.

### Macro and Micronutrients Content

Shoots and roots were rinsed in sterile water and dried to constant weight at 60°C. The samples were then weighed, ground and stored in hermetic plastic containers for the subsequent chemical analyses.

The contents of P, K, Ca, Mg, Na, S, Fe, Zn, Mn, Cu, Cr, Al, Ni, and Pb were quantified by inductively coupled plasma optical emission spectrometry (ICP-OES), after digestion with HNO_3_ (Merck) and H_2_O_2_ (Merck) in an open digestion system (Peters, 2005). ICP conditions were: plasma flow: 8.0 L⋅min^-1^, auxiliary gas 0.70 L⋅min^-1^ and carrier gas 0.55 L⋅min^-1^. The nutrient uptake of all macro and micronutrients were determined based on the plant dry matter multiplied by the nutrient concentration.

Elemental composition for carbon and nitrogen were determined on the bulk samples using ∼2 mg of dry plant tissues (shoot and roots). These analyses were performed by a Finnigan Delta V Advantage Thermal Isotopic Mass Spectrometer with Conflo IV and Thermo Scientific Flash 2000 Organic Elemental Analyzer. Triplicate analyses were carried out for every 10 samples with ∼95% of reproducibility. The detection limits for C and N were 0.05 and 0.02%, respectively.

### Leaf Gas Exchange, SPAD Values and Chlorophyll a Fluorescence

Leaf gas exchange was measured with a Li-Cor 6400 portable photosynthesis system (Li-Cor Inc., Lincoln, NE, United States), with external [CO_2_] supply of 400 mL⋅L^-1^, and an external light source at 1,000 μmol⋅m^-2^⋅s^-1^ photosynthetic photon flux density (PPFD) on a set of four leaves [recently mature leaves (fully sun-exposed)] per treatment (with four replicates each) from 08:00 to 10:00 h. Leaf gas exchange measurements included net photosynthesis (*A*), stomatal conductance (*g*_*s*_), transpiration rate (*E*), intrinsic water use efficiency (iWUE [*A*/*g*_*s*_]), internal/external CO_2_ concentration ratio (*C*_*i*_/*C*_*a*_) and the ratio of *A* to internal CO_2_ concentration (*A*/*C*_*i*_). After leaf gas exchange measurements, six SPAD values (leaf greenness) sampled from the same area, using the SPAD-502 Chlorophyll Meter (Minolta Co. Ltd., Osaka, Japan) were averaged. Fluorescence induction kinetics were measured with high temporal resolution during a saturation pulse using a non-modulated fluorimeter model pocket PEA (Plant Efficiency Analyser, Hansatech, King’s Lynn, Norfolk, United Kingdom). Excitation intensity was 3,500 μmol photons m^-2^⋅s^-1^ with red light of 650 nm for 3 s. From the fluorescence induction signal from 10 μs to 3 s the instrument determines initial (*F*_*o*_) and maximum (*F*_*m*_) fluorescence and the variable fluorescence (*F*_*v*_) at specified time intervals. Furthermore, it calculates specific parameters such as the potential quantum yield of PSII (*F*_*v*_/*F*_*m*_), the performance index (PI) [performance index (potential) for energy conservation from exciton to the reduction of intersystem electron acceptors]. *F*_*v*_/*F*_*m*_ and PI were measured on the same non-detached leaves used for gas exchange measurements between 08:00 and 10:00 h. The leaves were dark-adapted for ca. 30 min. with leaf clips (Plant Efficiency Analyser, Hansatech, King’s Lynn, Norfolk, United Kingdom) so that all reaction centers of PSII acquired an ‘open’ status, and heat loss was minimal (Strasser et al., 2000; Strasser and Tsimilli-Michael, 2001; Strasser et al., 2010). PI was calculated according to the equation (Strasser and Tsimilli-Michael, 2001; Strasser et al., 2010):

(1) $$PI=1-\frac{\frac{F_{0}}{F_{m}}}{\frac{M_{0}}{V_{j}}}\cdot \frac{F_{m}-F_{0}}{F_{0}}\cdot \frac{1-V_{j}}{V_{j}}$$

where *F*_0_ is fluorescence intensity at 50 ms; Fm is maximal fluorescence intensity; *V*_*j*_ is relative variable fluorescence at 2 ms, calculated as *V*_*j*_ = (*F*_*j*_ -*F*_0_)/(*F*_*m*_ - *F*_0_), in which *F*_*j*_ is the fluorescence intensity at step *j* (at 2 ms); and *M*_0_ is the initial slope of fluorescence kinetics, which can be derived from the equation:

(2) $$M_{0}=4\cdot \frac{F300\mathrm{\mu s}-F_{0}}{F_{m}-F_{0}}$$

### Membrane Isolation

Membrane vesicles were isolated from *O. sativa* roots using differential centrifugation, essentially as described by Giannini and Briskin (1987), with minor modifications. About 5 g of *O. sativa* roots were homogenized using a mortar and pestle in 2 mL g^-1^ (fresh weight) of ice-cold buffer containing: 250 mM sucrose, 10% (v/v) glycerol, 0.5% (v/v) PVP (PVP-40, 40 kD), 5 mM EDTA, 0.3% (w/v) BSA and 0.1 M Tris-HCl buffer, pH 7.6. Just prior to use, 150 mM KCl, 3.3 mM DTT, 1 mM PMSF and 1 mM benzamidine were added to the buffer. The homogenate was strained through four layers of cheesecloth and centrifuged at 1,500 *g* for 15 min. The supernatant was centrifuged once more at 10,000 *g* for 20 min and then at 100,000 *g* for 45 min. The pellet was resuspended in a small volume of ice-cold buffer containing 10 mM Tris-HCl, pH 7.6, 10% (v/v) glycerol, 1 mM EDTA, 1 mM DTT, 1 mM PMSF and 1 mM benzamidine. All preparative steps were performed at 4°C. The vesicles were frozen under liquid N_2_ and stored at -70°C until use. Protein concentrations were determined by the method of Bradford (1976).

### Determination of H^+^-ATPase and H^+^-PPase Hydrolytic Activity

H^+^-ATPase and H^+^-PPase hydrolytic activities were determined colorimetrically by measuring the release of *P*_*i*_ (Fiske and Subbarow, 1925). The reaction media contained 50 mM Tris-HCl pH 6.5, 3 mM MgSO_4_, 100 mM KCl, 0.2 mM NaMoO_4_ and 1 mM ATP or PPi. The reaction was started by addition of protein (30 μg⋅mL^-1^) and stopped with ice-cold 5% (w/v) trichloracetic acid after 30 min of incubation at 25°C. Specific inhibitors such as 0.2 mM Na_3_VO_4_ and 5 nM concanamycin a, were used to determine the hydrolytic activities of P-H^+^-ATPase and V-H^+^-ATPase, respectively. PPi hydrolysis, performed by H^+^-PPase, was determined through its dependency on K^+^.

### H^+^ Gradient Monitoring

ATP and PPi-dependent H^+^ transport across membranes was measured as the initial rate of fluorescence quenching of 9-amino-6-chloro-2-methoxyacridine (ACMA) at 25°C in a fluorimeter (model F-3010, Hitachi, Tokyo) using a protocol adapted by Façanha and de Meis (1998). The excitation wavelength was set at 415 nm and the emission wavelength was set at 485 nm. The reaction medium contained 10 mM Tris-HCl pH 6.5, 2 μM ACMA, 5 mM MgSO_4_, 100 mM KCl and 1 mM ATP or PPi. The reaction was initiated by the addition of 30 μg⋅mL^-1^ of membrane vesicles. The addition of 20 mM NH_4_Cl was used to show a recovery of the fluorescence that indicated a collapse of the preliminarily formed H^+^ gradient. Specific inhibitors such as 0.2 mM Na_3_VO_4_ and 5 nM concanamycin a, were used to determine the H^+^ pumping activities of P-H^+^-ATPase and V-H^+^-ATPase, respectively. H^+^-PPase pumping activity was determined through its dependence on K^+^.

### Statistical Analysis

The experiment was arranged in randomized block designs with three biological replicates. Results were statistically analyzed by two-way ANOVA and when a factor or any interaction between factors was deemed statistically significant, we performed pairwise comparisons by means of a *t*-test and correcting the corresponding results for multiple comparisons using Tukey’s test at *p* ≤ 0.05. All analyses were conducted using the program GraphPad Prism 7.0 using a 5% significance level for hypothesis testing.

## Results

### Growth Responses and Fungal Colonization

**FIGURE 1:**
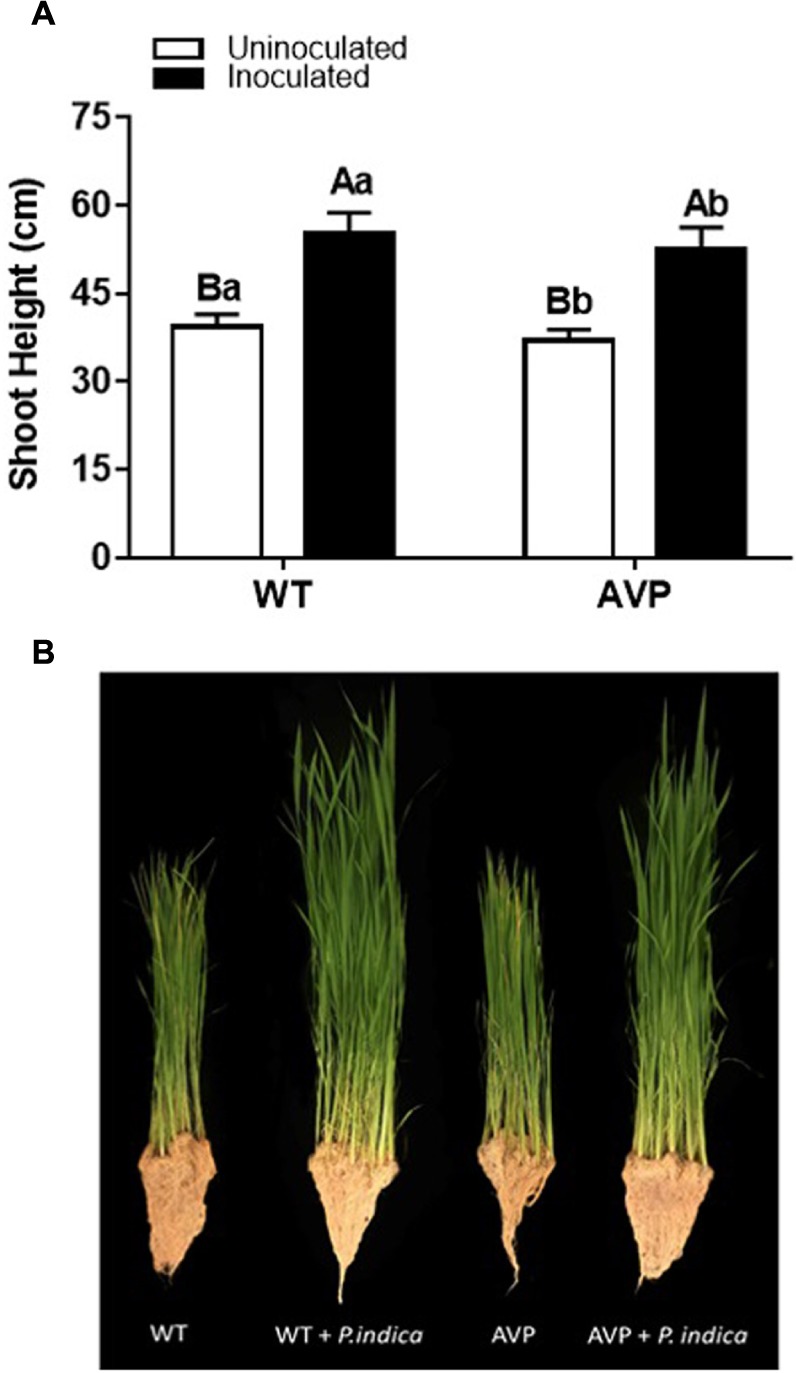
**(A)** Plant height of transgenic rice overexpressing (AVP) or not (WT) vacuolar H^+^-PPase, inoculated or not with the endophytic fungus *P. indica*. **(B)** Visualization of plant growth. The data was analyzed by two-way ANOVA combined with Tukey’s test. For each rice genotype (WT or AVP), bars followed by the same uppercase letter, in different inoculation conditions (uninoculated or inoculated), are not significantly different by Tukey’s test at *p* < 0.05. For each inoculation condition (uninoculated or inoculated), bars followed by the same lowercase letter, at the same genotype, are not significantly different at *p* < 0.05 (*n* = 10).

**FIGURE 2:**
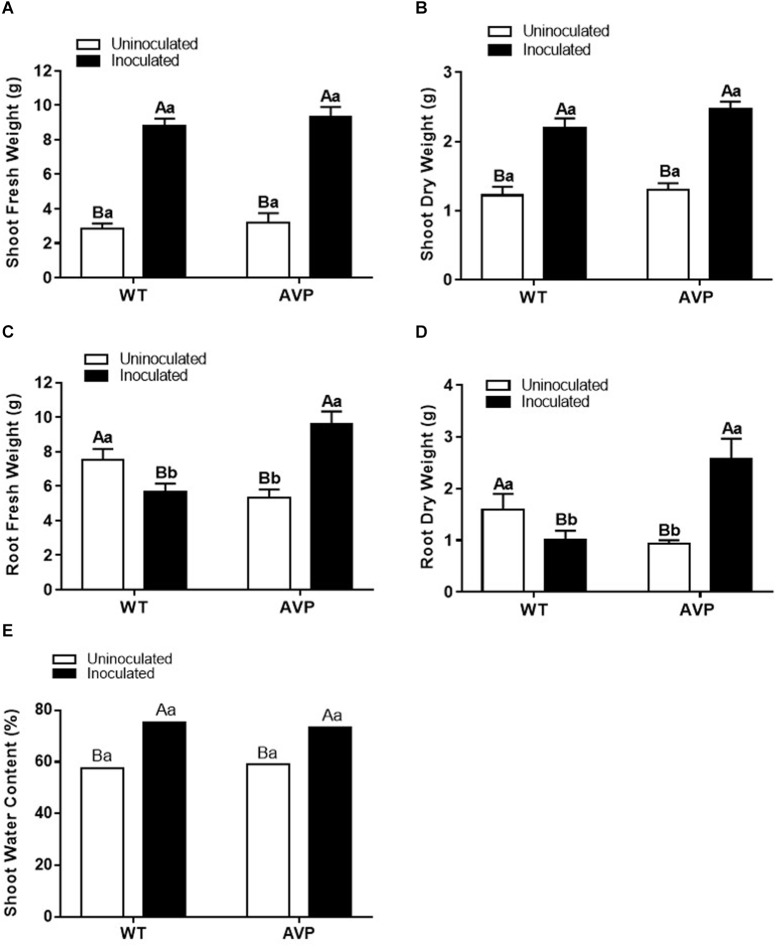
Shoot fresh matter **(A)**, shoot dry matter **(B)**, root fresh matter **(C)**, root dry matter **(D)**, and shoot water content **(E)** of transgenic rice overexpressing (AVP) or not (WT) the vacuolar H^+^-PPase, inoculated or not with the endophytic fungus *P. indica*. The data was analyzed by two-way ANOVA combined with Tukey’s test. For each rice genotype (WT or AVP), bars followed by the same uppercase letter, in different inoculation conditions (uninoculated or inoculated), are not significantly different by Tukey’s test at *p* < 0.05. For each inoculation condition (uninoculated or inoculated), bars followed by the same lowercase letter, at the same genotype, are not significantly different at *p* < 0.05 (*n* = 10).

The WT and AVP rice plants inoculated with *P. indica* showed a significant (*P* < 0.0001) increase in all growth parameters, in comparison with the uninoculated plants, with the exception of the root fresh and dry weight of the wild-type genotype (Figure 1, 2).

Fungal inoculation increased shoot height by 40%, in both WT and AVP, compared with uninoculated plants (Figure 1A). There was a significant (*P* < 0.0001) increase in shoot height of WT plants in comparison with AVP, regardless of the fungal presence (Figure 1A). Both genotypes inoculated with *P. indica* showed enhanced shoot water content, which was 31.04 and 23.77% higher, in WT and AVP, than in the uninoculated genotypes (Figure 1B). Plant inoculation with *P. indica* stimulated shoot fresh matter by 210.15 and 193.63% and shoot dry matter by 80.82 and 91.69% of WT and AVP plants, respectively (Figure 2A,B). There were no differences in shoot fresh and dry matter when comparing WT and AVP uninoculated or WT and AVP inoculated. Root fresh and dry matter was only stimulated by the fungus in the transgenic plants (Figure 2C,D). WT uninoculated showed higher root fresh and dry matter than AVP uninoculated plants, whereas the opposite occurred in WT- and AVP- inoculated plants. Inoculated rice plants showed significantly higher shoot water content than uninoculated plants (Figure 2E).

**FIGURE 3:**
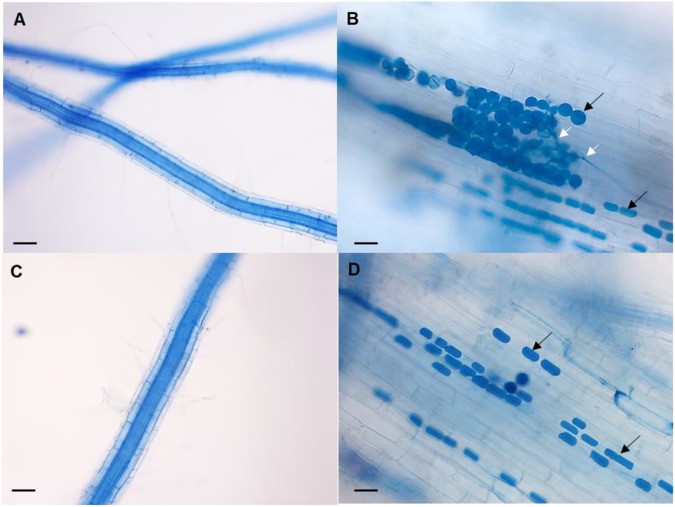
Representative micrograph of the fungal colonization in rice roots of WT uninoculated **(A)**, WT inoculated with *P. indica* **(B)**, AVP uninoculated **(C)**, and AVP inoculated with *P. indica* **(D)**. Colonized roots show the presence of *P. indica* spores (black arrows) and the presence of hyphae germinating from the spores (white arrows). Bars represent 40 μM **(A,C)** and 20 μM **(B,D)**. Roots were stained with trypan blue 0.02%.

Roots were evaluated microscopically for root colonization, but no chlamydospores were observed in WT and AVP uninoculated plants (Figure 3A,C). Root colonization was 40% in WT inoculated and 11.11% in AVP inoculated plants (Table 1). Numerous chlamydospores and hyphae emerging from the spores were observed in both inoculated genotypes, however, inoculated wild-type roots showed more rounded chlamydospores (Figure 3B), while most spores of transgenic inoculated roots were rectangular (Figure 3D).

**Table 1:** Assessment of *P. indica* colonization in inoculated WT or AVP rice roots.

Treatments	WT	Wt + *P. indica*	AVP	AVP + *P. indica*
Colonization (%)	–	40.0 ± 1.04a	–	11.1 ± 0.95b


*Different letters in each column shows statistically significant at p < 0.05 according to Tukey’s test. Values are showed as means (n = 5).*

### Leaf Gas Exchange and Chlorophyll Fluorescence Parameters

**FIGURE 4:**
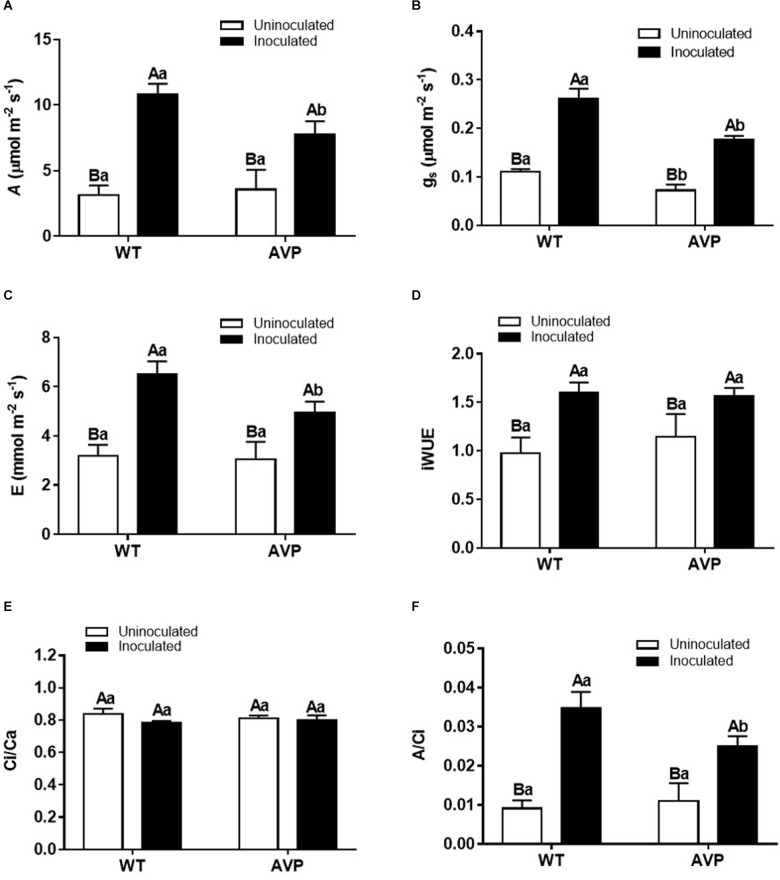
Net carbon assimilation (*A*) **(A)**, stomatal conductance (*g_*s*_*) **(B)**, transpiration (E) **(C)**, intrinsic water use efficiency (iWUE) **(D)**, internal/external carbon concentration (*C*i/*C*_a_) **(E)**, and carboxylation efficiency (*A*/*C*_*i*_) **(F)** in leaves of transgenic rice overexpressing (AVP) or not (WT) the vacuolar H^+^-PPase, inoculated or not with the endophytic fungus *P. indica*. The data was analyzed by two-way ANOVA combined with Tukey’s test. For each rice genotype (WT or AVP), bars followed by the same uppercase letter, in different inoculation conditions (uninoculated or inoculated), are not significantly different by Tukey’s test at *p* < 0.05. For each inoculation condition (uninoculated or inoculated), bars followed by the same lowercase letter, at the same genotype, are not significantly different at *p* < 0.05 (*n* = 4).

Net carbon assimilation (*A*) was significantly higher in plants inoculated with *P. indica* than the control ones (Figure 4A), being stimulated by 234.71 and 117.12% in inoculated WT and AVP plants, respectively. There was no significant difference in *A* between uninoculated WT and AVP plants, but among inoculated genotypes, *A* was lower in AVP than in WT plants (Figure 4A).

Stomatal conductance (*g*_*s*_) and transpiration rate (*E*) responded similarly (Figure 4B,C). Both E and g_*s*_ were significantly higher in WT and AVP plants inoculated compared with the respective controls (Figure 4B,C). Inoculation of transgenic rice with *P. indica* did not alter *g*_*s*_ relative to the wild-type inoculation, even though *E* was reduced in inoculated AVP. Intrinsic water use efficiency (iWUE) was stimulated by 64.36 and 74.66% in WT and AVP inoculated with *P. indica*, relative to the uninoculated plants (Figure 4D).

Despite the variations in *A, g*_*s*_, and *E*, the ratio between internal and external CO_2_ concentration (*C*_*i*_/*C*_*a*_) was not altered in all treatments (Figure 4E). The observed carboxylation efficiency response profile (Figure 4F) was similar to that of C assimilation (Figure 4A).

**FIGURE 5:**
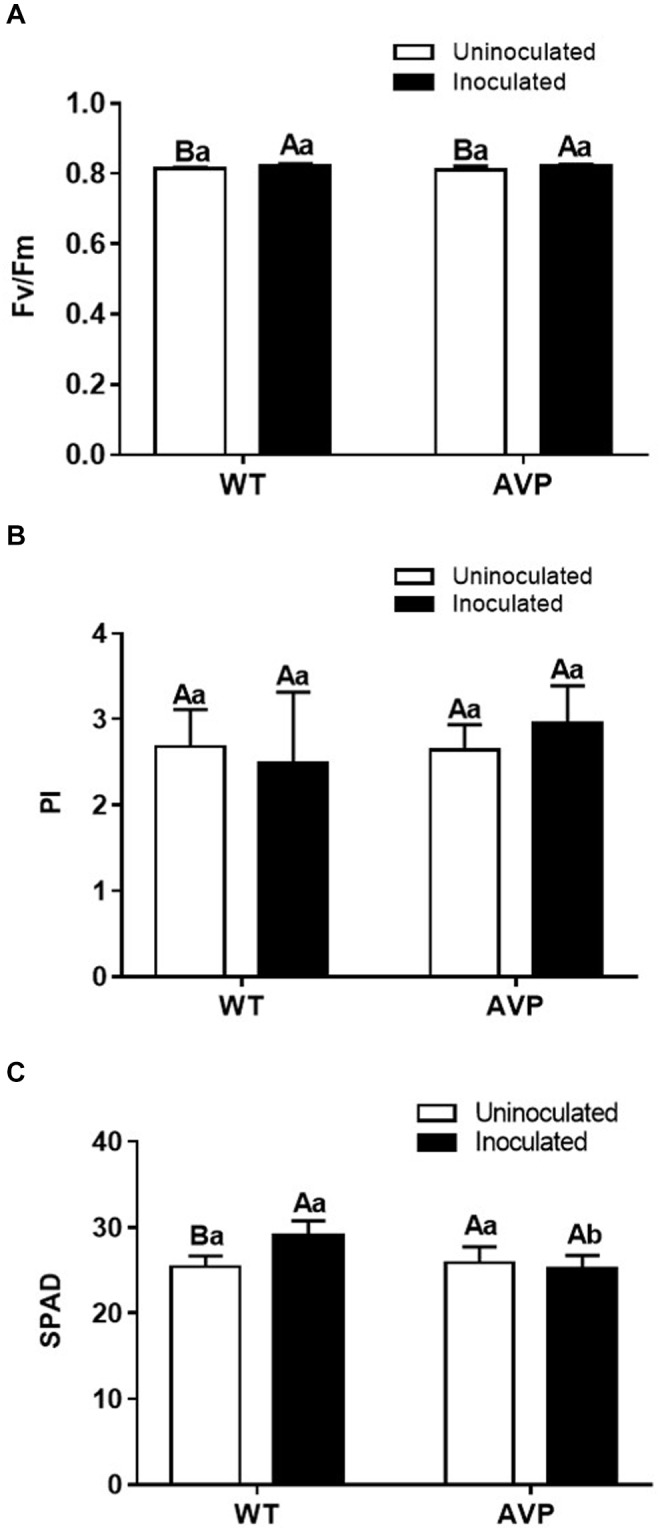
Potential quantum yield of PSII (*F*_*v*_/*F*_*m*_) **(A)**, performance index (PI) **(B)**, and relative chlorophyll content (SPAD reading, leaf greenness) **(C)** in leaves of transgenic rice overexpressing (AVP) or not (WT) the vacuolar H^+^-PPase, inoculated or not with the endophytic fungus *P. indica*. The data was analyzed by two-way ANOVA combined with Tukey’s test. For each rice genotype (WT or AVP), bars followed by the same uppercase letter, in different inoculation conditions (uninoculated or inoculated), are not significantly different by Tukey’s test at *p* < 0.05. For each inoculation condition (uninoculated or inoculated), bars followed by the same lowercase letter, at the same genotype, are not significantly different at *p* < 0.05 (*n* = 4).

In all treatments the potential quantum yield of PSII (*F*_*v*_/*F*_*m*_) values were within the range considered normal, varying from 0.82 to 0.75 (Figure 5A). PI values were not altered in all treatments (Figure 5B). Fungal inoculation only enhanced chlorophyll content in the leaves of WT plants, indeed the inoculation of AVP with *P. indica* reduced their chlorophyll content compared to WT inoculated plants (Figure 5C).

### Hydrolytic Activity of H^+^-ATPases and H^+^-PPases

**FIGURE 6:**
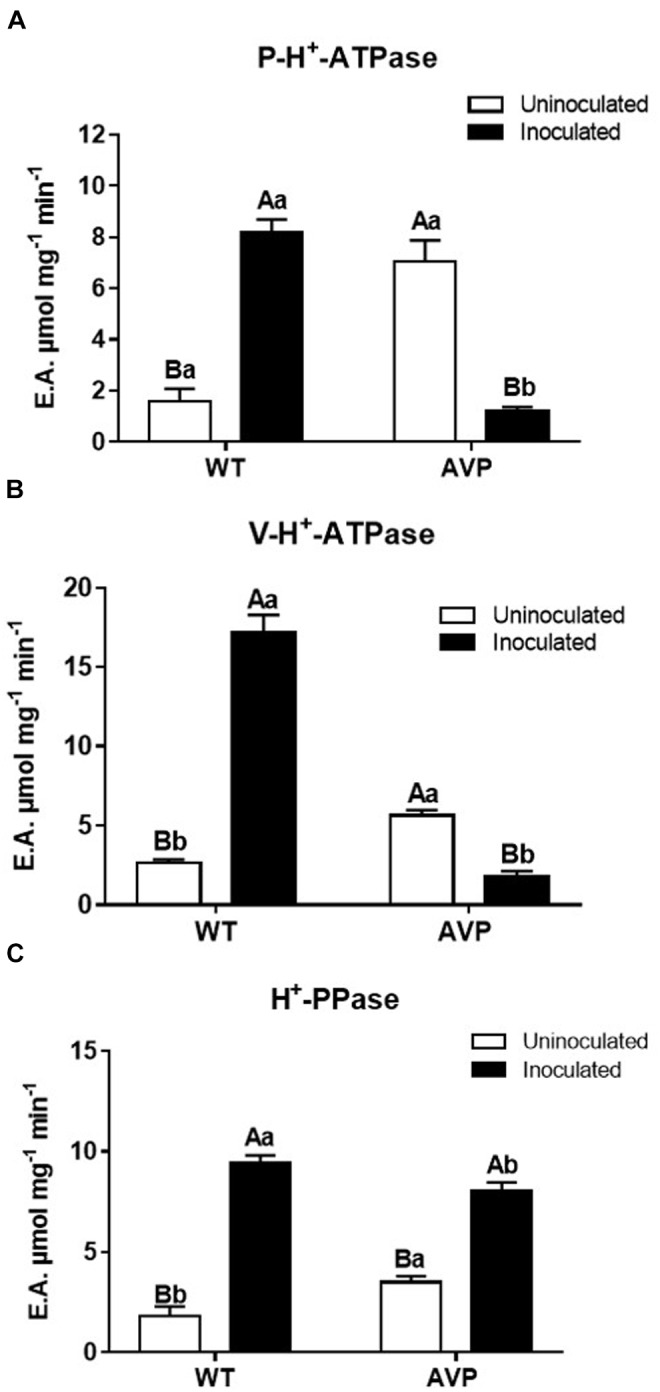
Specific hydrolytic activity of plasma membrane H^+^-ATPases (P-H^+^-ATPase) **(A)**, vacuolar H^+^-ATPases (V-H^+^-ATPase) **(B)**, and vacuolar H^+^-Pyrophosphatases (H^+^-PPase) **(C)** in the microsomal fraction of transgenic rice roots overexpressing (AVP) or not (WT) the vacuolar H^+^-PPase, inoculated or not with the endophytic fungus *P. indica*. The data was analyzed by two-way ANOVA combined with Tukey’s test. For each rice genotype (WT or AVP), bars followed by the same uppercase letter, in different inoculation conditions (uninoculated or inoculated), are not significantly different by Tukey’s test at *p* < 0.05. For each inoculation condition (uninoculated or inoculated), bars followed by the same lowercase letter, at the same genotype, are not significantly different at *p* < 0.05 (*n* = 3).

The hydrolytic activity of P-H^+^-ATPases in inoculated WT plants was 415.25% higher than in uninoculated WT plants (Figure 6A). However, inoculation of transgenic plants caused and inhibition of 83.14% in P-H^+^-ATPases hydrolytic activity, relative to uninoculated AVP (Figure 6A). A substantial inhibition of P-H^+^-ATPases hydrolytic activity was also observed in AVP-inoculated, compared to WT-inoculated plants. However, P-H^+^-ATPases hydrolytic activity of uninoculated AVP plants was significantly higher than that of uninoculated WT plants (Figure 6A).

The hydrolytic activity profile of the V-H^+^-ATPases was similar to that observed in P-H^+^-ATPases (Figure 6B): inoculated WT plants showed V-H^+^-ATPases hydrolytic activity 483% higher than uninoculated WT plants. However, inoculation of the transgenic plants caused an inhibition of V-H^+^-ATPases’ hydrolytic activity by 68.5%, relative to uninoculated AVP plants. As in P-H^+^-ATPases, uninoculated AVP plants showed significantly higher ATP hydrolysis than uninoculated WT plants (Figure 6B).

H^+^-PPases hydrolytic activity in WT and AVP plants inoculated with *P. indica* was 548.38 and 186.96% higher than in the respective controls (Figure 6C). Uninoculated transgenic rice showed significantly higher H^+^-PPase hydrolytic activity than WT uninoculated rice. Comparing the inoculated genotypes, H^+^-PPase hydrolysis was significantly inhibited in transgenic plants (Figure 6C).

### H^+^ Transport of H^+^-ATPases and H^+^-PPases

**FIGURE 7:**
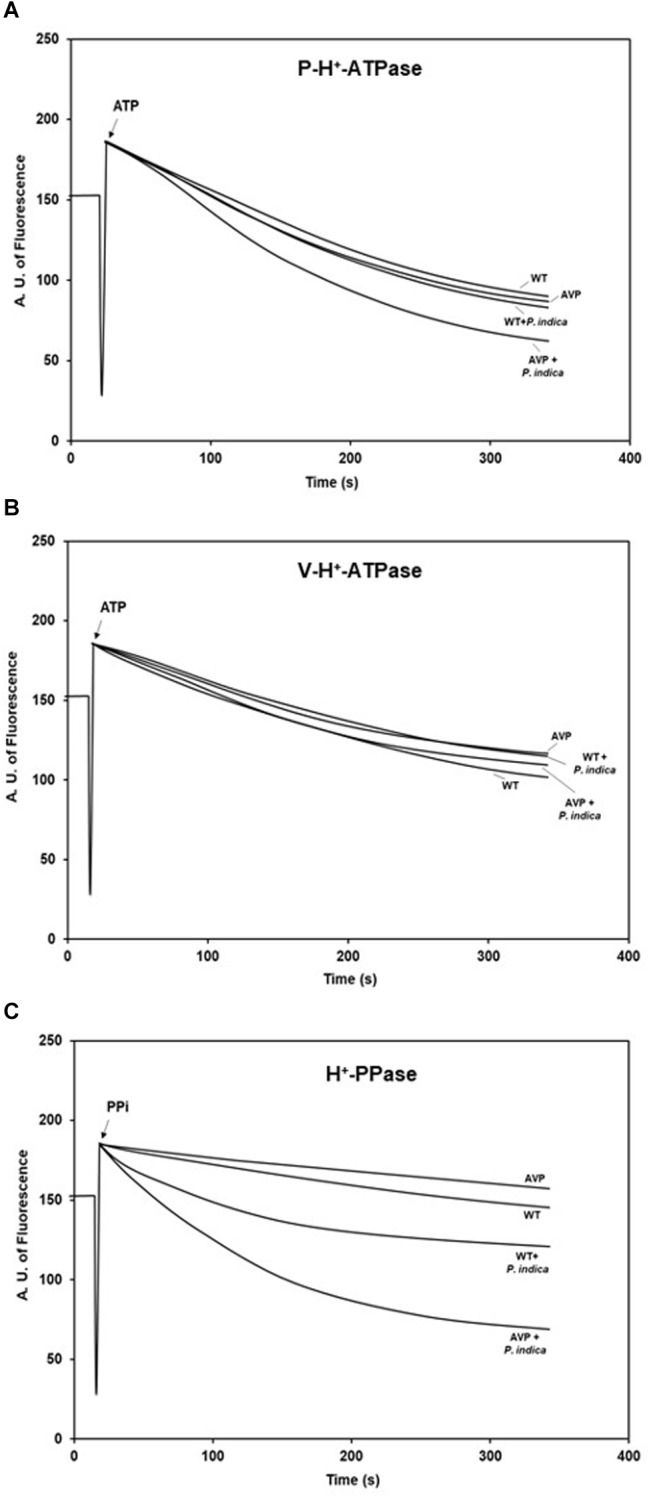
H^+^ pumping activities of plasma membrane H^+^-ATPases (P-H^+^-ATPase) **(A)**, vacuolar H^+^-ATPases (V-H^+^-ATPase) **(B)**, and vacuolar H^+^-Pyrophosphatases (H^+^-PPase) **(C)** in the microsomal fraction of transgenic rice roots overexpressing (AVP) or not (WT) the vacuolar H^+^-PPase, inoculated or not with the endophytic fungus *P. indica*. H^+^ translocation across membrane vesicles was monitored by the fluorescence quenching of ACMA in the presence of 60 μg protein of membrane vesicles. The reaction was started by addition of 1 mM ATP for P-H^+^-ATPase and V-H^+^-ATPase and 1mM PPi for H^+^-PPase and stopped with 20 mM NH_4_Cl.

P and V-H^+^-ATPases had similar patterns of H^+^ transport initial velocity (*V*_0_) (Figure 7A,B and Supplementary Table S1). There were no significant differences in the *V*_0_ between the inoculated and uninoculated genotypes, with or without the fungus (Figure 7A,B and Supplementary Table S1). Nevertheless, the *V*_0_ of H^+^-PPases was stimulated by 358.90 and 703.45% in inoculated WT and AVP plants, respectively, compared to the control ones (Figure 7C and Supplementary Table S1). There were no significant differences in the *V*_0_ of H^+^-PPases between uninoculated WT and AVP, or between inoculated WT and AVP.

**FIGURE 8:**
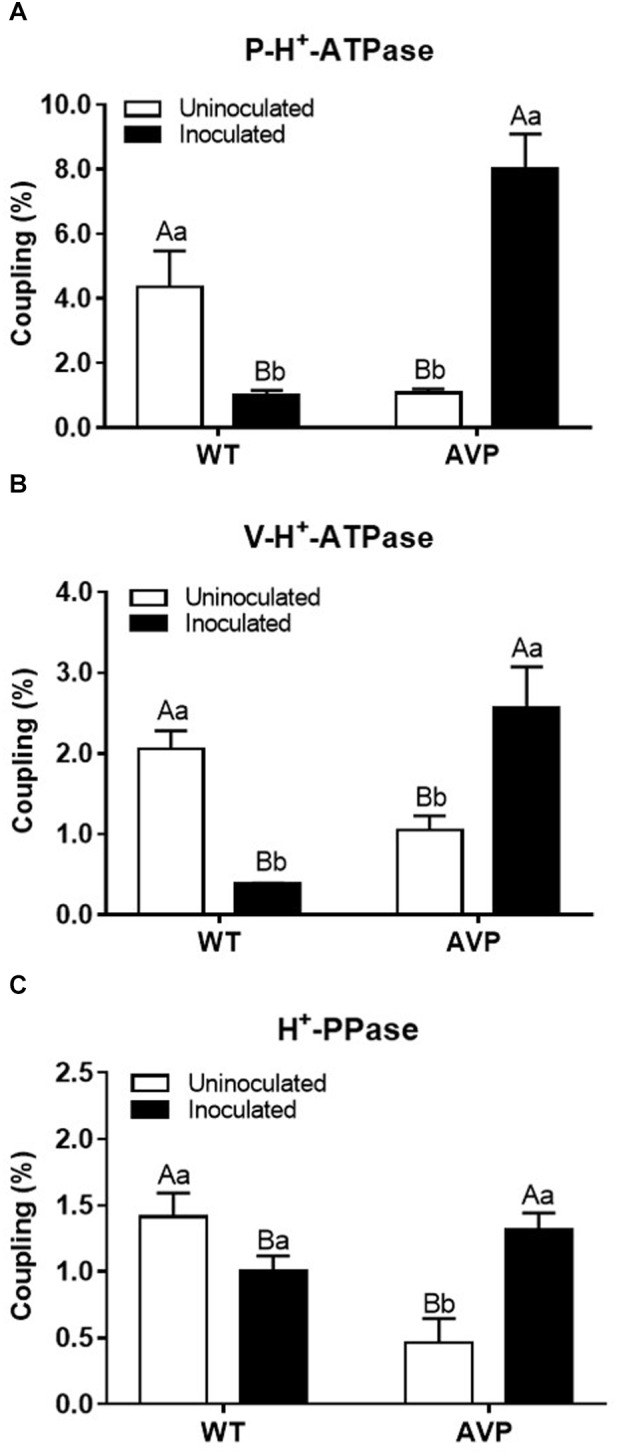
Coupling ratio (*V*_0_ of H^+^ pumping/ATP or PPi hydrolysis) of plasma membrane H^+^-ATPases (P-H^+^-ATPase) **(A)**, vacuolar H^+^-ATPases (V-H^+^-ATPase) **(B)**, and vacuolar H^+^-Pyrophosphatases (H^+^-PPase) **(C)** in the microsomal fraction of transgenic rice roots overexpressing (AVP) or not (WT) the vacuolar H^+^-PPase, inoculated or not with the endophytic fungus *P. indica*. The data was analyzed by two-way ANOVA combined with Tukey’s test. For each rice genotype (WT or AVP), bars followed by the same uppercase letter, in different inoculation conditions (uninoculated or inoculated), are not significantly different by Tukey’s test at *p* < 0.05. For each inoculation condition (uninoculated or inoculated), bars followed by the same lowercase letter, at the same genotype, are not significantly different at *p* < 0.05 (*n* = 3).

The highest difference in maximum fluorescence (ΔMF) of P-H^+^-ATPase was found in AVP inoculated plants (Figure 8A and Supplementary Table S1). Inoculation of WT and AVP with *P. indica* caused no significant changes in V-H^+^-ATPases ΔMF; but when comparing the uninoculated genotypes, V-H^+^-ATPases ΔMF was inhibited by 21.40% in the transgenic rice plants (Figure 8B and Supplementary Table S1). In both genotypes, H^+^-PPase ΔMF was substantially stimulated by fungal inoculation, compared to the respective controls (Figure 8C and Supplementary Table S1). Comparing uninoculated genotypes, H^+^-PPase ΔMF was inhibited by 42.33% in AVP plants; however, between inoculated genotypes, H^+^-PPase ΔMF was stimulated by 27.66% in AVP rice plants (Figure 8C and Supplementary Table S1).

### Shoot Nutrient Content

The observed shoot nutrient contents are showed in Table 2. Fungal inoculation increased the contents of N, P, and K relative to the controls (Table 2). Regarding the uninoculated rice genotypes, only N content was higher in AVP plants, while there was no difference in P and K contents. However, when comparing the inoculated rice genotypes, the N and P contents were inhibited in the AVP plants, while there was no significant difference in K content (Table 2).

**Table 2:** Shoot nutrient content of transgenic rice overexpressing (AVP) or not (WT) the vacuolar H^+^-PPase, inoculated or not with the endophytic fungus *P. indica*.

Nutrients		Treatments			
	
		WT	WT + *P. indica*	AVP	AVP + *P. indica*
		**g kg^-1^**			

**Macronutrients**	N	11.48 Bb	18.74 Aa	12.58 Ba	15.34 Ab
	P	0.52 Ba	2.68 Aa	0.38 Ba	2.02 Ab
	K	21.36 Ba	31.22 Aa	22.80 Ba	29.02 Aa
	Ca	5.38 Aa	5.36 Aa	5.90 Aa	4.92 Ba
	Mg	2.16 Ba	6.54 Aa	1.63 Aa	2.10 Ab
	Na	1.15 Aa	0.81 Aa	1.29 Aa	1.15 Aa
	S	1.76 Ba	4.12 Aa	1.43 Bb	2.33 Ab
	C	372.74 Aa	387.42 Aa	372.49 Aa	391.41 Aa
	N/P	22.21 Ab	6.99 Ba	33.31 Aa	7.61 Ba

		**mg kg^-1^**			

**Macronutrients**	Ni	5.48 Aa	3.02 Bb	4.39 Aa	4.46 Aa
	Cu	6.09 Ba	7.49 Aa	3.30 Bb	4.72 Ab
	Zn	38.75 Aa	51.36 Aa	33.41 Ba	65.95 Aa
	Cr	13.66 Bb	53.78 Aa	40.14 Aa	38.75 Ab
	Fe	974.67 Aa	569.00 Ba	626.67 Ab	558.33 Aa
	Mn	556.8 Ab	312.9 Ba	621.6 Aa	217.3 Bb
	Al	2293.33 Aa	919.33 Ba	1118.67 Ab	766.33 Aa
	Pb	0.99 Ab	0.85 Ab	2.37 Aa	1.57 Ba


*The data was analyzed by two-way ANOVA combined with Tukey’s test. For each rice genotype (WT or AVP), means followed by the same uppercase letter, in different inoculation conditions (uninoculated or inoculated), are not significantly different by Tukey’s test at p < 0.05. For each inoculation condition (uninoculated or inoculated), means followed by the same lowercase letter, at the same genotype, are not significantly different at p < 0.05 (n = 3).*

Shoot Ca content was lower in AVP inoculated than in AVP uninoculated plants, and there were no significant differences among the other treatments. Inoculation with *P. indica* increased Mg content in WT compared to the control, but decreased Mg content in transgenic rice compared to the inoculated wild-type rice (Table 2). WT and AVP inoculated plants showed higher S contents than their respective controls, also WT rice plants inoculated or not, showed higher S contents than AVP plants, inoculated or not, respectively (Table 2). There were no significant differences in Na and C contents of all treatments.

Similarly, the Fe and Al contents of the wild-type genotype were decreased by fungal inoculation, and also lower in AVP than WT uninoculated plants (Table 2). Zn content was only higher in the inoculated than uninoculated transgenic rice plants. On the other hand, Ni content was lower in inoculated WT than in the uninoculated wild-type (Table 2). WT and AVP plants inoculated with *P. indica* showed lower Mn contents than uninoculated genotypes. Among uninoculated genotypes, Mn content was higher in AVP plants, but among inoculated genotypes, Mn content was higher in WT plants (Table 2).

Cr and Pb contents were higher in WT inoculated than in WT uninoculated plants, but it was lower in transgenic inoculated than in wild-type inoculated rice plants (Table 2). Regardless of the genotype, plants inoculated with *P. indica*, had higher Cu contents than the respective controls, and in general AVP plants had lower Cu contents than WT plants (Table 2).

### Root Nutrient Content

P, K, Na, and S contents of WT roots inoculated with *P. indica* were significantly higher than in WT uninoculated and AVP inoculated roots (Table 3). Mg content was significantly inhibited in AVP inoculated compared to WT inoculated and to AVP uninoculated plants. Fungal inoculation enhanced N content in WT, but decreased it in AVP plants, compared to the respective controls. Among the uninoculated genotypes, N content was highest in AVP roots, however, among the inoculated genotypes, N content was lowest in the transgenic rice roots (Table 3). Root C content was lower in inoculated AVP than in WT inoculated and AVP uninoculated plants, but comparing uninoculated genotypes, C content was higher in AVP roots (Table 3).

**Table 3:** Root nutrient content of transgenic rice overexpressing (AVP) or not (WT) the vacuolar H^+^-PPase, inoculated or not with the endophytic fungus *P. indica*.

Nutrients		Treatments			
	
		WT	WT + *P. indica*	AVP	AVP + *P. indica*
		**g kg^-1^**			

**Macronutrients**	N	6.22 Bb	7.78 Aa	7.81 Aa	4.72 Bb
	P	0.42 Ba	0.95 Aa	0.26 Aa	0.37 Ab
	K	4.64 Ba	7.66 Aa	3.92 Aa	3.04 Ab
	Ca	0.85 Ba	1.31 Aa	0.77 Aa	0.50 Bb
	Mg	1.33 Aa	1.87 Aa	1.33 Aa	1.12 Bb
	Na	3.12 Ba	5.63 Aa	2.04 Aa	2.71 Ab
	S	1.12 Ba	2.09 Aa	0.66 Aa	0.83 Ab
	C	176.45 Ab	190.46 Aa	239.69 Aa	126.56 Bb
	N/P	15.23 Ab	8.60 Aa	30.86 Aa	12.96 Ba

		**mg kg^-1^**			

**Macronutrients**	Ni	6.24 Bb	13.63 Aa	15.95 Aa	8.76 Bb
	Cu	5.88 Aa	4.98 Aa	5.20 Aa	4.56 Aa
	Zn	15.91 Aa	17.21 Aa	14.44 Ba	25.89 Aa
	Cr	23.50 Bb	34.28 Aa	42.61 Aa	18.68 Bb
	Fe	7330.67 Aa	5366.33 Ba	6385.33 Aa	2399.67 Bb
	Mn	71.77 Aa	53.1 Ba	82.8 Aa	24.23 Bb
	Al	31702.7Aa	26717.3 Aa	26207.7 Aa	14928.7 Bb
	Pb	4.45 Ba	12.65 Aa	4.11 Aa	3.1 Ab


*The data was analyzed by two-way ANOVA combined with Tukey’s test. For each rice genotype (WT or AVP), means followed by the same uppercase letter, in different inoculation conditions (uninoculated or inoculated), are not significantly different by Tukey’s test at p < 0.05. For each inoculation condition (uninoculated or inoculated), means followed by the same lowercase letter, at the same genotype, are not significantly different at p < 0.05 (n = 3).*

Roots inoculated with *P. indica* had decreased Fe and Mn contents, compared to the respective controls (Table 3). Among inoculated genotypes, AVP roots showed reduced content of Fe, Mn, Ni, Cr, Al, and Pb. There were no significant differences between Cu contents of all treatments (Table 3). Root Zn content was higher in AVP inoculated than in AVP uninoculated plants. On the other hand, Al, Ni, and Cr contents were lower in inoculated than uninoculated transgenic roots. WT inoculated roots contained higher contents of Ni, Cr, and Pb than to uninoculated WT roots. Among uninoculated genotypes, AVP displayed higher Ni and Cr contents than WT roots (Table 3).

## Discussion

In the present study we analyzed the interaction of transgenic rice plants overexpressing the *AVP1* gene with the PGPM *P. indica*, using morphological, ecophysiological, biochemical and nutritional characteristics (Figure 1, 2, 4–8 and Tables 2, 3), and found the fungus to be generally positive for plant development (Prajapati et al., 2008; Bagheri et al., 2013; Jogawat et al., 2013).

The changes in plant morphology and biomass due to interaction with *P. indica* are compatible with plant answers to increased root levels of auxin, produced by the fungus (Sirrenberg et al., 2007; Vadassery et al., 2008) or by the plant after being stimulated by the fungus (Hua et al., 2017). Moreover, *P. indica* has been shown to up-regulate the expression of AUX1 and to down-regulate the expression of PIN3 in Chinese cabbage roots (Lee et al., 2011; Dong et al., 2013), which could represent a possible pathway for chemical crosstalk between the plant, even the transgenic one, and the fungus. The drastic reduction of AVP1OX root colonization by *P. indica* (Table 1) may also be related with the strong acidification of its rhizosphere relative to the WT plants, since AVP1OX plants can acidify the medium to ∼pH5.2, and *P. indica* ’s optimum growth is at pH 6.5 (Yang et al., 2007; Paez-Valencia et al., 2013). Soil pH is known to have considerable influence on spore number and germination, hyphal growth and root colonization of AMF, which varies greatly according to the fungal species (Coughlan et al., 2000; Van Aarle et al., 2002). It has also been commonly reported that root colonization by AMF is generally less in low than in high pH soils (Clark, 1997).

*Piriformospora indica* stimulated rice, WT and AVP1OX plants, gas exchange (*A, A*/*C*_*i*_, *g*_*s*_, and *E*) and photochemical efficiency (*F*_*v*_/*F*_*m*_ and SPAD reading in WT) (Figure 4, 5) as previously described by Hussin et al. (2017) and Ghorbani et al. (2018). Photosynthetic carbon assimilation (*A*) in inoculated plants was increased due to higher stomatal conductance (*g*_*s*_). In WT plants, *A* increased due to higher *g*_*s*_ (stomatal factors), and *F*v/*F*m and SPAD reading (non-stomatal factors). In addition, *P. indica* increased *F*v/*F*m in both control and osmotic-stressed plants of rice plants, and in fact, an association between *P. indica* and rice seedlings provided a multifaceted protection to rice plants under osmotic stress (-0.295 MPa) (Saddique et al., 2018). Under abiotic stress, *P. indica* was also found to stabilize chlorophyll in rice leaves (Abadi and Sepehri, 2016). In consequence, it increases the maximum quantum yield of PSII (*F*v/*F*m) (Vahabi et al., 2016; Shahabivand et al., 2017). It is worth mentioning that only a few studies analyzed gas exchange parameters in transgenic plants with overexpression of H^+^-PPase gene (AVP1) of Arabidopsis (Park et al., 2005; Raza et al., 2016). It is interesting that the improving effect of *P. indica* inoculation on intrinsic water use efficiency (iWUE, Figure 4D) was detectable at the level of tissue water content (Figure 1). It has been shown that one of the *YUCCA* genes (*YUCCA6*), plays an important role in maintaining auxin levels in *Arabidopsis* and many other plants, such as tomato, maize, rice and petunia, and is involved in mediating plant drought tolerance and auxin levels (Ke et al., 2015). The endogenous pool of IAA in plants may be altered by the acquisition of IAA secreted by soil microorganisms (Mathesius et al., 1998; Bianco et al., 2014; Ansari et al., 2017). Auxin overproduction, or accumulation, may also lead to adjustments in the synthesis or distribution of other hormones, such as ABA, known to control water balance through its effect on stomata and the expression of osmotic-stress-tolerance genes (Defez et al., 2017). From this perspective, *P. indica* may improve plant water balance by reducing water loss, and promoting water acquisition, through the induction of an improved root system and mycelium access to water outside the reach of the root system. The same mechanism of improved water uptake is commonly observed in plants inoculated with arbuscular mycorrhizal fungi, which also results in higher *A, g*_*s*_, *E* (Porcel et al., 2015) and nutrient use efficiency.

*Piriformospora indica*-mediated growth promotion of WT and AVP1OX rice plants was associated with increased macro and micro nutrient uptake, especially P (Tables 2, 3). This is compatible with the up-regulation of high affinity phosphate transporters such as PiPT in the hyphae (Shahollari et al., 2007; Yadav et al., 2010), and of transporters such as PhT1-1 to PhT1-5 in roots, as previously described for *A. thaliana* and maize (Johnson et al., 2014). It has also been demonstrated that in symbiosis with roots of *Brassica napus, Serendipita indica* (*P. indica*) promotes the mobilization and uptake of P from inorganic sources, through the combined effects of stimulation of P-solubilizing phosphatase activity, production of organic acids and stimulation of BnPht1;4 and BnACP5 genes, under P limitation conditions (Meiyan et al., 2018). Uptake and assimilation of N are also enhanced by *P. indica* colonization of several host plants. The fungus may enhance NADH-dependent nitrate reductase (Nia2) activity in roots, as observed in *A. thaliana* and tobacco, increasing N assimilation, and promoting nitrate uptake from the soil (Sherameti et al., 2005) and plant growth. N uptake can also affect chlorophyll content, which can directly influence the photosynthetic capacity of plants (Coruzzi and Bush, 2001; Dordas and Sioulas, 2008). The effects of *P. indica* inoculation on N and P acquisition drastically decreased the N/P ratio of the plant tissues of both WT and AVP1OX plants, contradicting the stable leaf nutrient concentration hypothesis. This highlights the relevance of symbiosis in systems response to global changes, since the relationships between metabolically N and P contents, their allocations in different plant tissues and plant photosynthetic capacity are often used to predict future C sequestration of eco- and agro-systems (Tang et al., 2018).

The over-expression of the *A. thaliana* vacuolar pyrophosphatase AVP1 in rice plants affected nutrient acquisition and allocation to a much lesser extent than *P. indica* inoculation (Tables 2, 3). In fact, inoculated AVP1OX had lower *A, E, g*s, and *A*/*C*_*i*_ than inoculated WT plants (Figure 4), which may be the result of a higher *P. indica* interaction with the WT relative to the AVP1OX plants, detected through smaller root biomass allocation (Figure 2); higher colonization rates and a higher number of intraradical *P. indica* spores (Figure 3 and Table 1). This higher carbon investment in fungal structures may also explain why *P. indica*-inoculated WT plants accumulate less biomass than inoculated AVP1OX plants despite having higher photosynthetic activities.

It has been proposed that the increased nutrient uptake consistently observed in AVP1 transgenic plants occurs due to its phenotype of larger root biomass and greater rhizosphere acidification (Heuer et al., 2017), however, these were not observed in the conditions of this study. Therefore, the difference in nutrient uptake between inoculated genotypes could be related to the impaired fungal colonization observed in AVP1OX rice roots, which showed only 11% of colonized roots, whereas WT plants showed approximately fourfold more colonized roots (Table 1). It is thus likely that AVP1OX plants, with less colonized roots, would have a less robust root system than WT inoculated plants, thus, the effect promoted by the over-expression of the AVP1 gene, would not be enough to promote higher root growth than the fungus. This is demonstrated by previous studies of Prajapati et al. (2008), which observed a fourfold higher root dry weight of 45 days old rice plants inoculated with *P. indica* when compared to uninoculated rice, and Yang et al. (2007), which observed only a twofold higher root dry weight of 45 days old AVP1OX rice plants when compare to WT rice plants. The higher colonization of WT rice plants in relation to AVP1OX plants could also be related to the increased plant growth and nutrient absorption due to an efficient absorption, transport and mobilization of nutrients from the soil and further efficient translocation to the aerial parts of the plant (Shahollari et al., 2007).

The over-expression of the *A. thaliana* vacuolar pyrophosphatase AVP1 results in root proliferation and increased P-H^+^-ATPase-mediated rhizosphere acidification (Yang et al., 2007). However, we only observed these features when comparing inoculated plants (Figure 7). Also, the reduced hydrolytic activity of those pumps observed in inoculated AVP1OX rice (Figure 6) demonstrates an enhanced efficiency of H^+^ pumps’ coupling (Figure 8), as previously observed in plants exposed to stress (Gaxiola et al., 2001; Venancio et al., 2014; Schilling et al., 2017). This observation supports the idea that although plant inoculation with PGPM results in increased plant development, the inoculation is associated with a moderated biotic stress, which activates defense mechanisms and stimulates the plant (Shrivastava et al., 2018; Xu et al., 2018). *P. indica* initially colonizes living cells through invagination of the plasma membrane, maintaining the cell organelles intact. But, this biotrophic phase is followed by a cell death-associated colonization phase, revealed to occur through induction of endoplasmic reticulum (ER) stress and suppression of unfolded protein response, resulting in ER swelling and activation of a γVPE/caspase 1–like-mediated cell death program, preceded by vacuolar collapse (Schäfer and Kogel, 2009; Qiang et al., 2012). Colonization with *P. indica* was also shown to reduce the availability of free sugars and amino acids to the root tip, to change host hormone homeostasis and to be dependent on efficient suppression of plant immune responses (Schäfer et al., 2009; Daneshkhah et al., 2018).

There is a huge interest in the use of transgenic plants with superior yields and improved traits in agriculture. However, there are serious concerns over their potential impact on non-target soil microorganisms, especially those that form symbioses and are beneficial to plants (Wolfenbarger and Phifer, 2000; Lynch et al., 2004; Liu et al., 2005), as observed in transgenic tobacco and maize plants, which had significantly reduced root colonization of the mycorrhizal fungus *Glomus mosseae* (Vierheilig et al., 1995; Medina et al., 2003; Castaldini et al., 2005). Our results indicate that for plants inoculated with *P. indica*, the phenotypes of the WT and AVP1OX plants were very similar; with the WT plants presenting higher height, fungal colonization, ecophysiological parameters, H^+^ pumps hydrolytic activity and nutrient content (especially that of P). On the one hand, this shows that symbiotic interactions may promote crop growth and performance as much as transgenic plants in a much faster way. On the other hand, they justify the importance of studying the interaction of transgenic plants with soil microorganisms, to assess whether the effects of these microorganisms would indeed be beneficial to such plants when in symbiosis. To better understand the regulatory pathways that underlie the colonization of *P. indica* with transgenic AVP1 plants, further studies involving molecular analysis, proteomics or transcriptomics approaches are necessary.

## Data Availability

All datasets generated for this study are included in the manuscript and/or the Supplementary Files.

## Author Contributions

AB and AR conceived and designed the experiments. AB and SS performed the experiments. AB, SS, and KR performed the photosynthetic analysis. AB and CMC performed the bioenergetic analysis. AB and CR performed the nutritional analysis. AB and AR wrote the manuscript. EC reviewed and helped to write the photosynthesis results. CC, JM, and AV reviewed and helped to write the manuscript. CR helped to write the nutritional results. All authors contributed to the discussion and approved the final manuscript.

## Conflict of Interest Statement

The authors declare that the research was conducted in the absence of any commercial or financial relationships that could be construed as a potential conflict of interest.
