# Supplementary material for: Inoculation With Piriformospora indica Is More Efficient in Wild-Type Rice Than in Transgenic Rice Over-Expressing the Vacuolar H+-PPase
Source: Front Microbiol. 2019 May 15;10:1087. doi: 10.3389/fmicb.2019.01087 (PMC6530341; doi:10.3389/fmicb.2019.01087)
Supplement: Supplementary file 1 [file Table_1.DOCX]

**Table S1.** Comparison of H^+^ transport initial velocity (V_0_) and the difference in maximum fluorescence (ΔMF) of P-H^+^-ATPase, V-H^+^-ATPase and H^+^-PPase of transgenic rice overexpressing (AVP) or not (WT) the vacuolar H^+^-PPase, inoculated or not with the endophytic fungus *P. indica*. The data was analyzed by two-way ANOVA combined with Tukey`s test. For each rice genotype (WT or AVP), means followed by the same uppercase letter, in different inoculation condition (uninoculated or inoculated), are not significantly different by Tukey`s test at *p*<0.05. For each inoculation condition (uninoculated or inoculated), means followed by the same lowercase letter, at the same genotype, are not significantly different at *p*<0.05 (n = 3).

|  | **P-H^+^-ATPase** | | **V-H^+^-ATPase** | | **H^+^-PPase** | |
| --- | --- | --- | --- | --- | --- | --- |
|  | V_0_ (%) | ΔMF (%) | V_0_ (%) | ΔMF (%) | V_0_ (%) | ΔMF (%) |
| WT | 6.56 Aa | 47.36 Aa | 5.51 Aa | 39.58 Aa | 2.36 Ba | 18.52 Ba |
| WT+*P. indica* | 8.11 Aa | 45.55 Ab | 6.01 Aa | 36.36 Aa | 10.83 Aa | 38.00 Ab |
| AVP | 7.57 Aa | 50.54 Ba | 5.37 Aa | 31.11 Ab | 1.45 Ba | 10.68 Bb |
| AVP+*P. indica* | 9.38 Aa | 58.33 Aa | 5.06 Aa | 35.71 Aa | 11.65 Aa | 48.51 Aa |
